# Shear Behaviour and Calculation Methods of Bearing-Shear Connectors for Prefabricated Steel–Concrete Composite Beams

**DOI:** 10.3390/ma16134616

**Published:** 2023-06-26

**Authors:** Zhichao Zheng, Yang Zou, Yaling Chou, Fengjiang Qin, Fengmin Chen, Jin Di, Zhigang Zhang

**Affiliations:** 1 Key Laboratory of New Technology for Construction of Cities in Mountain Area, School of Civil Engineering, Chongqing University, Chongqing 400030, China; zhichaozheng1@163.com (Z.Z.); dijin@cqu.edu.cn (J.D.); zhangzg@cqu.edu.cn (Z.Z.); 2 State Key Laboratory of Mountain Bridge and Tunnel Engineering, Chongqing Jiaotong University, Chongqing 400074, China; zouyang@cqjtu.edu.cn; 3 Key Laboratory of Disaster Prevention and Mitigation in Civil Engineering, Lanzhou University of Technology, Lanzhou 730050, China; chouyaling@lzb.ac.cn; 4 China Railway Changjiang Transport Design Group Co., Ltd., Chongqing 401121, China; chenfengmin@ccrdi.cmhk.com

**Keywords:** bearing-shear connectors, numerical analysis, failure mechanism, shear resistance, slip modulus, calculation formulae

## Abstract

The bearing-shear connector (B-SC) is a newly developed connector that exhibits excellent shear behaviour and is easy to process. However, research on the application of B-SCs as substitutes for grouped studs in prefabricated steel–concrete composite beams is rare, and systematically studying their shear behaviour is necessary. Thus, a refined numerical model was developed to study the shear behaviour of the B-SCs. The numerical model, validated by push-out tests, was conducted to analyse the stress of the B-SCs and concrete slab during loading and to explore the failure mechanism of B-SCs. Then, a parametric study was performed to identify the key factors influencing the shear behaviour of the B-SCs. The concrete strength, and the thickness and the tensile strength of the shear plate were found to significantly influence the shear behaviour of B-SCs. According to the experiments and numerical analysis, calculation formulae for the ultimate shear resistance and slip modulus were proposed.

## 1. Introduction

Prefabricated steel–concrete composite bridges have been increasingly used in many countries owing to their advantages, such as good economy, simple construction, and convenient disassembly and replacement in the later stages [1,2,3,4]. The main components, concrete decks and steel beams, are prefabricated in the factory and then transported to the site for assembly, which dramatically shortens the construction period and minimises traffic interference. The mechanical performance of prefabricated composite structures is significantly affected by the behaviour of shear connectors [5].

Common shear connectors suitable for prefabricated composite bridges include studs, bolts, and section-steel connectors. Studs are the most common choice for prefabricated composite bridges. However, grouped studs were required to be compactly arranged in bridges with large shear force at the S-C (steel beam–concrete slab) interface, which would increase the size of the reserved holes in precast concrete decks [6,7,8] that not only increase the difficulty of formwork but also cause the reinforcing bars and grouped studs to interfere with each other in the reserved holes. In response to the above-mentioned problems, multiple studies [9,10,11,12] suggest using large-diameter studs. Push-out tests have demonstrated that the application of large-diameter studs improves the shear resistance per stud but also increases the risk of concrete slab splitting [12]. Compared with studs, bolts are not only more convenient to install and disassemble but also have better fatigue strength because they do not require welding [13]. The shear resistance of bolts is close to that of studs of the same size, but their slip modulus is inferior to that of studs [13,14,15,16]. Channel connectors [17,18,19,20] and C-connectors [21,22,23] have the advantages of easy processing, small reserved holes, and high shear resistance. However, their initial slip modulus is lower and they have different mechanical properties in opposite directions [20]. Additionally, owing to the existence of a steel flange, concrete is prone to cracking during loading. T-perfobond connectors have the advantages of high shear resistance and slip modulus [24]. However, the failure of T-perfobond connectors is often accompanied by the brittle crushing failure of concrete. The plastic deformation of T-perfobond connectors cannot be large enough to redistribute the load in actual structures owing to their poor deformation ability.

**Figure 1:**
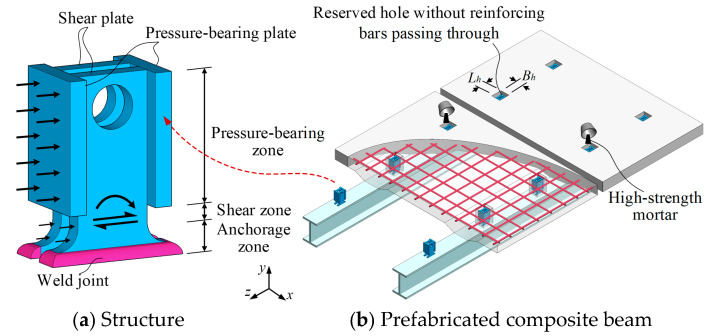
Bearing-shear connector (B-SC).

The bearing-shear connectors (B-SCs), composed of pressure-bearing plates and shear plates, have a simple structural design, as illustrated in Figure 1 [25]. Push-out and beam tests are often performed to investigate the shear behaviour of connectors. However, studying the shear behaviour of connectors through a large number of full-scale tests is difficult because of time and cost. Finite element (FE) modelling, as an effective alternative, has been used by many researchers to investigate the shear behaviour of connectors [26,27,28,29,30]. Therefore, this study aims at establishing an accurate FE model capable of providing further insight into the shear behaviour of the B-SCs. Then, the FE model, verified by experimental results, was applied to analyse the effect of the concrete strength, and the thickness and tensile strength of the shear plate on the shear behaviour of the B-SCs. Finally, based on the results of push-out tests and FE analysis, calculation formulae for the ultimate shear resistance and slip modulus of the B-SCs were proposed.

## 2. Summary of Push-Out Tests

**Figure 2:**
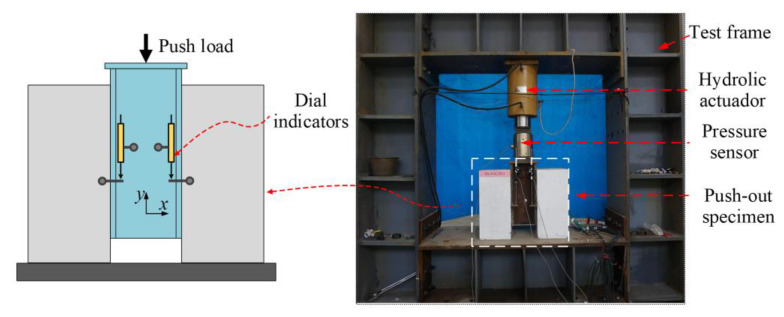
Test setup.

**Figure 3:**
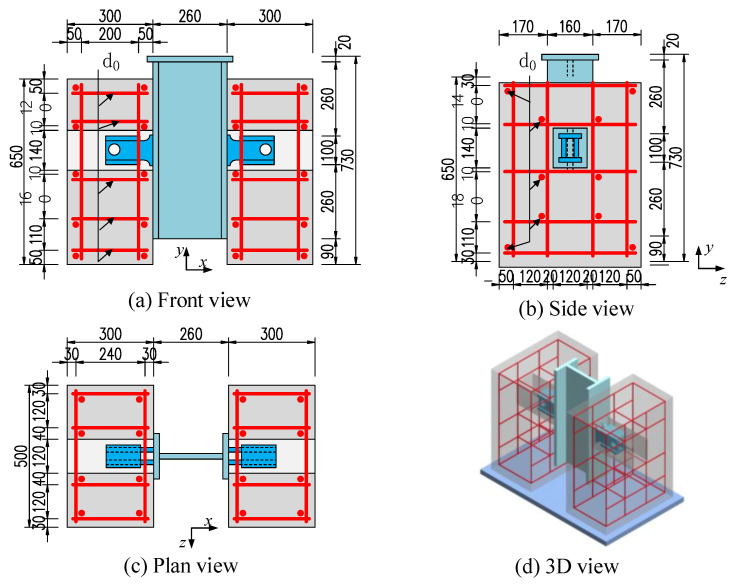
Geometry and dimensions of the specimens (mm).

Fifteen push-out specimen tests were performed by Zou [25] to study the effect of the geometry of B-SCs on their shear behaviour. Figure 2 and Figure 3 show the test setup and geometric dimensions of the specimens manufactured in accordance with Eurocode 4 [31], respectively.

**Figure 4:**
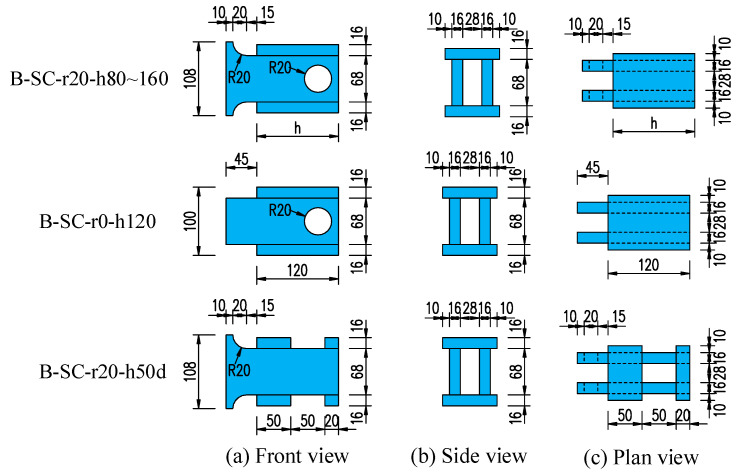
Details of the B-SCs (mm).

Each push-out specimen consisted of a 620 mm high H-steel beam (260 × 160 × 20 × 20 mm), two precast concrete slabs (500 mm × 300 mm × 650 mm), and two B-SCs. The pressuring-bearing plates and shear plates were made of Q345 and Q420, respectively [32]. Full penetration welding and fillet welding were used between the B-SCs and the steel beams, shear plates and pressure-bearing plates, respectively. The weld leg length of fillet welding was 16 mm. Figure 4 shows the structural details of the B-SCs.

Each precast concrete slab had a reserved hole (120 × 140 mm) to accommodate the B-SCs and non-shrinkage high-strength mortar was cast into the reserved hole to achieve the composite action. Take the specimen B-SC-r20-h120 as an example; “r20” denotes the radius of the chamfer of the shear plate to 20; “h120” denotes the height of the pressure-bearing plate to 120.

## 3. Finite-Element Modelling

### 3.1. Geometry Model and Mesh

**Figure 5:**
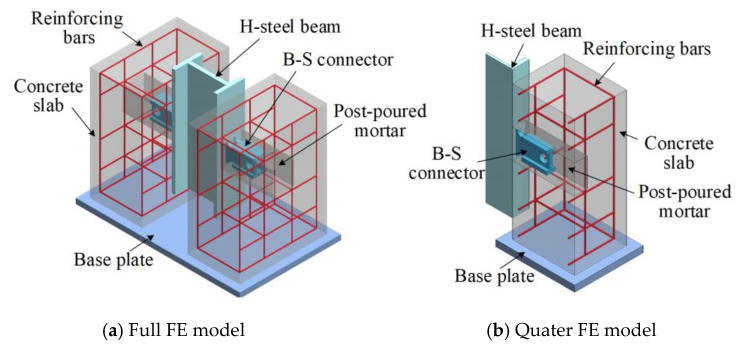
Geometry model of the push-out specimens.

The general static-analysis method available in ABAQUS [33] was applied to model push-out tests [27,28,29]. A quarter FE model was developed for the biaxial symmetry of the specimens, as shown in Figure 5. The FE model considered the material and geometric nonlinearities. Taking the specimen B-SC-r20-h120 as an example, the complete process of FE modelling was introduced in detail.

**Figure 6:**
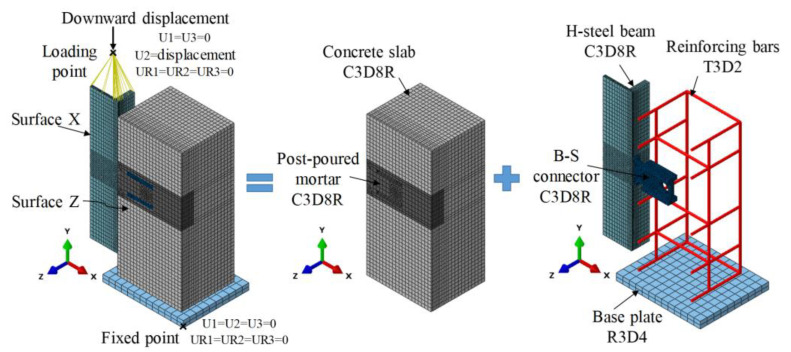
FE model and mesh.

As shown in Figure 6, three types of elements were applied for meshing. The B-SC, concrete slab, post-poured mortar, and steel beam were meshed with solid elements (C3D8R), which not only prevented shear-locking difficulties but also provided reasonable accuracy when compared with other element types [27,28,29]. The reinforcing bars were meshed using truss elements (T3D2) [27,28,29]. The rigid element (R3D4) was meshed the for base plate. The mesh size varied with the geometric size and importance of different parts. For example, the global and local seed sizes for the concrete slab and steel beam were 15 and 5 mm, respectively. To maintain the continuity of the element sizes and improve the convergence of the FE model, the mesh sizes of the B-SC and reinforcing bars were 5 and 15 mm, respectively.

### 3.2. Boundary Conditions and Loading Protocol

Symmetric boundary conditions were considered in the quarter FE model. As presented in Figure 6, all nodes on Surfaces X and Z were restrained from moving in the X and Z direction, respectively. The base plate was assumed fixed. A downward enforced displacement in the Y-direction was applied at the “Loading point”.

### 3.3. Contact Modelling

**Figure 7:**
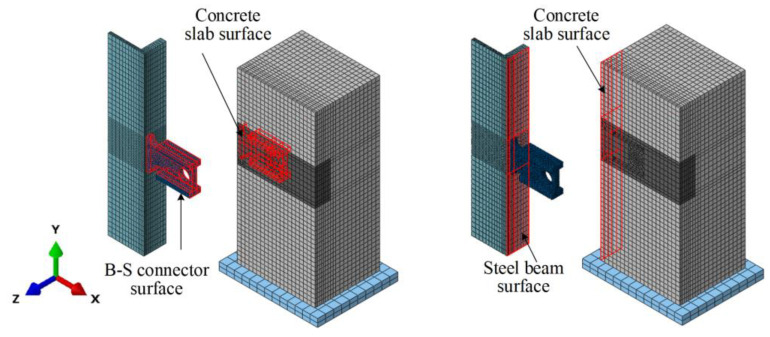
Contact modelling.

In this study, two types of contact properties were employed for the interaction. First, contact interactions were used at the interface of the above-mentioned components, as shown in Figure 7. The normal “hard” contact and tangential “penalty” frictional formulation were considered for the first contact interaction. The friction coefficient between the steel beam and concrete slab was 0.6 [27,28], and that between the other components was 0.25 [28,29]. The reinforcing bars were “embedded” in their surrounding concrete.

**Figure 8:**
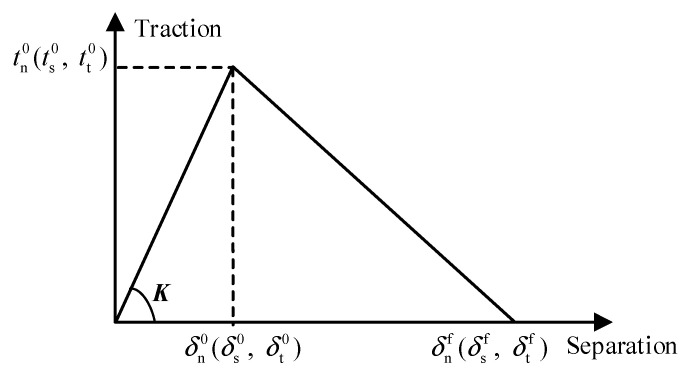
Typical traction and separation response.

Second, the bonding force of the S-C interface significantly impacts the initial slip modulus of the connectors in push-out tests [27,34]. Therefore, the influence of the bonding force of the S-C interface on the shear performance of the B-SC should be considered. In addition to the first-contact property, surface-based cohesive behaviour was adopted to model the bonding force between the steel beam and concrete slab [34]. The bilinear traction–separation relationship was used to model the cohesive behaviour, as illustrated in Figure 8 [28,34,35]. The traction–separation model initially assumes a linear elastic behaviour, followed by the initiation and evolution of damage [35]. The uncoupled traction-separation type is given by Equation (1):

(1) $$t=\left\{\begin{array}{l} t_{n}\\ t_{s}\\ t_{t} \end{array}\right\}=\left[\begin{array}{lll} K_{\mathrm{nn}} & 0 & 0\\ 0 & K_{\mathrm{ss}} & 0\\ 0 & 0 & K_{\mathrm{tt}} \end{array}\right]\left\{\begin{array}{l} \delta_{n}\\ \delta_{s}\\ \delta_{t} \end{array}\right\}=K\delta$$

According to the findings of a previous study [34], the parameters of cohesive behaviour were applied as follows: *K*_nn_ was considered as 0.05 *E*_cm_, *K*_ss_, and *K*_tt_ were considered as 0.05 *G*_cm_, where *E*_cm_ and *G*_cm_ are the elastic and shear modulus of concrete, respectively. *K*_nn_, *K*_ss_, and *K*_tt_ are the elastic stiffness of the cohesive contact property [35]. The quadratic-stress criterion shown in Equation (2) was used as the damage-initiation criterion for cohesive behaviour. The parameters associated with the damage to cohesive behaviour were determined as follows: $t_{n}^{0}=0.05$, $t_{s}^{0}=t_{t}^{0}=0.3$ [34,35] and $\delta_{n}^{f}=0.8$ mm [34]; *t*_n_, *t*_s_ and *t*_t_ are the tractions of the cohesive contact property [35].

(2) $${\left(\frac{\langle t_{n}\rangle }{t_{n}^{0}}\right)}^{2}+{\left(\frac{\langle t_{s}\rangle }{t_{s}^{0}}\right)}^{2}+{\left(\frac{\langle t_{t}\rangle }{t_{t}^{0}}\right)}^{2}=1$$

### 3.4. Material Models

#### 3.4.1. Concrete

**Figure 9:**
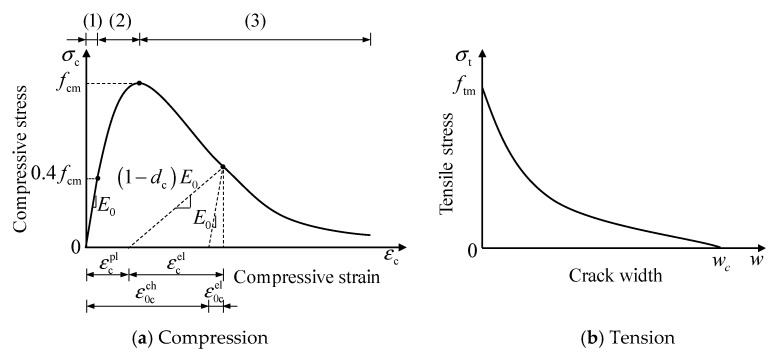
Concrete uniaxial behaviour.

“Concrete Damaged Plasticity” (CDP) was employed to model the uniaxial behaviour of concrete, as shown in Figure 9 [35]. The CDP assumes that the two primary failure modes of concrete are tensile cracking and compressive crushing, which are highly consistent with the failure modes of the concrete in these specimens.

Figure 9a,b present the uniaxial compression and tension behaviours of concrete, respectively. The stress–strain curve of the concrete under uniaxial compression is separated into three stages, as illustrated in Figure 9a. The first stage is linear ($0\leq \sigma_{c}\leq 0.4f_{\mathrm{cm}}$) [36].

(3) $$\sigma_{c\;(1)}=E_{\mathrm{cm}}\varepsilon_{c}$$

In Equation (3), *σ*_c_ and ε_c_ are the compressive stress and compressive strain of concrete, respectively; *f*_cm_ is the cylinder compressive strength of concrete. The cylinder compressive strength of concrete and non-shrinkage high-strength mortar were 44.5 MPa and 55.7 MPa, respectively. *E*_cm_ is the concrete elastic modulus, $E_{\mathrm{cm}}=E_{0}\alpha_{E}{\left(f_{\mathrm{cm}}/10\right)}^{1/3}$, $E_{0}=21.5$ GPa, and $\alpha_{E}=1.0$. *E*_0_ and *α*_E_ are the undamaged concrete elastic modulus and concrete aggregates factor, respectively. The second (ascending) stage is quadratic ($0.4f_{\mathrm{cm}}<\sigma_{c}\leq f_{\mathrm{cm}}$) [37].

(4) $$\sigma_{c\;(2)}=-\left(\frac{k\eta -\eta^{2}}{1+\left(k-2\right)\eta }\right)f_{\mathrm{cm}}$$

In Equation (4), $k=E_{\mathrm{cm}}\varepsilon_{\mathrm{cm}}/f_{\mathrm{cm}}$. The peak strain *ɛ*_cm_ corresponding to the peak stress *f*_cm_ was equal to 0.025 [36]. $\eta \left(=\varepsilon_{c}/\varepsilon_{\mathrm{cm}}\right)$ is a coefficient. The third (descending) stage considers the dependency of the specimen geometry to ensure almost mesh-independent simulation results [37,38]:

(5) $$\sigma_{c\;(3)}={\left(\frac{2+\gamma_{c}f_{\mathrm{cm}}\varepsilon_{\mathrm{cm}}}{2f_{\mathrm{cm}}}-\gamma_{c}\varepsilon_{c}+\frac{\varepsilon_{c}^{2}\gamma_{c}}{2\varepsilon_{\mathrm{cm}}}\right)}^{-1}$$

(6) $$\gamma_{c}=\frac{\pi^{2}f_{\mathrm{cm}}\varepsilon_{\mathrm{cm}}}{2{\left[\frac{G_{\mathrm{ch}}}{l_{\mathrm{ck}}}-0.5f_{\mathrm{cm}}\left(\varepsilon_{\mathrm{cm}}\left(1-b\right)+b\frac{f_{\mathrm{cm}}}{E_{0}}\right)\right]}^{2}}$$

In Equation (6), *G*_ch_ represents the crushing energy per unit area, $G_{\mathrm{ch}}={\left(f_{\mathrm{cm}}/f_{\mathrm{tm}}\right)}^{2}G_{f}$; *f*_tm_ is the tensile strength of concrete and is given in the literature [39,40]; *G*_f_ represents the fracture energy per unit area, $G_{f}=0.073f_{\mathrm{cm}}^{0.18}$ (N/mm) [38]. *l*_ck_ is the characteristic element length, which depends on the type of element and mesh size. For three-dimensional solid elements, *l*_ck_ is the cube root of the element volume [37]. The value of *b* ($\varepsilon_{c}^{\mathrm{pl}}/\varepsilon_{c}^{\mathrm{in}}$) was 0.7, assuming that the majority of the inelastic compressive strain remained after unloading [37]. $\varepsilon_{c}^{\mathrm{pl}}$ and $\varepsilon_{c}^{\mathrm{pl}}$ are the compressive plastic strain and compressive inelastic strain of concrete, respectively.

The tensile behaviour of concrete exhibited two distinct stages. When the principal tensile stress of concrete did not exceed its peak tensile stress, no cracks in the concrete were assumed, and uncracked concrete kept elastic under tension. For cracked concrete, ABAQUS expresses the tensile-softening behaviour of concrete in three ways: stress–strain, tensile stress-crack width, and fracture energy [35]. A nonlinear tensile stress-crack width equation was applied to express the tensile brittle behaviour of concrete in this study [39,40,41].

(7) $$\frac{\sigma_{t}}{f_{\mathrm{tm}}}=\left[1+{\left(c_{1}\frac{w}{w_{c}}\right)}^{3}\right]\exp\left(-c_{2}\frac{w}{w_{c}}\right)-\frac{w}{w_{c}}\left(1+c_{1}^{3}\right)\exp\left(-c_{2}\right)$$

**Figure 10:**
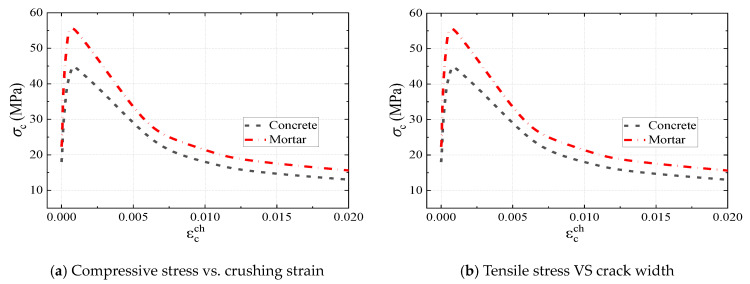
Material constitution of concrete.

In Equation (7), *c*_1_ = 3 and *c*_2_ = 6.93 [37,38], where *w*_c_ is the cracking width corresponding to the zero tensile stress and $w_{c}=5.14G_{f}/f_{\mathrm{tm}}$. The concrete-compression damage variable *d*_c_ and the tensile-damage variable *d*_t_ are used to express the deterioration of the concrete under compression and tension (Figure 10), respectively, and are given by Equations (8) and (9):

(8) $$d_{c}=1-\frac{1}{2+\alpha_{c}}\left[2\left(1+\alpha_{c}\right)\exp\left(-b_{c}\varepsilon_{c}^{\mathrm{ch}}\right)-\alpha_{c}\exp\left(-2b_{c}\varepsilon_{c}^{\mathrm{ch}}\right)\right]$$

(9) $$d_{t}=1-\frac{1}{2+\alpha_{t}}\left[2\left(1+\alpha_{t}\right)\exp\left(-b_{t}\varepsilon_{t}^{\mathrm{ck}}\right)-\alpha_{t}\exp\left(-2b_{t}\varepsilon_{t}^{\mathrm{ck}}\right)\right]$$

(10) $$b_{c}=\frac{1.97f_{\mathrm{cm}}}{G_{\mathrm{ch}}}l_{\mathrm{ck}}$$

(11) $$b_{t}=\frac{0.453{\left(f_{\mathrm{cm}}-8\right)}^{2/3}}{G_{f}}l_{\mathrm{ck}}$$

In Equations (8) and (9), $\alpha_{c}=7.873$, $\alpha_{t}=1$ $\varepsilon_{c}^{\mathrm{ch}}=\varepsilon_{c}-\sigma_{c}/E_{0}$, $\varepsilon_{t}^{\mathrm{ck}}=\varepsilon_{t}-\sigma_{t}/E_{0}$, $\varepsilon_{t}=\varepsilon_{\mathrm{tm}}-w/l_{\mathrm{ck}}$, where *α*_c_, *α*_t_, *b*_c_, and *b*_t_ are the dimensionless coefficients, *ε*_tm_ is the tensile peak strain of concrete. $\varepsilon_{c}^{\mathrm{ch}}$ and $\varepsilon_{t}^{\mathrm{ck}}$ are the compressive crushing strain and tensile cracking strain of concrete, respectively.

To accurately define the plastic-damage model of concrete, the following five additional parameters are required: dilation angle *ψ =* 37° [13]; flow potential eccentricity *ε* = 1; raio of biaxial to uniaxial compressive strength *σ*_bo_/*σ*_co_ = 1.16; ratio of *K* = 2/3, and viscosity parameter *μ* = 0.005 [34]. The application of the CDP yields an unsymmetric material-stiffness matrix. Thus, an unsymmetric matrix storage and solution scheme should be adopted in the step module to achieve an appropriate convergence rate in ABAQUS [35].

#### 3.4.2. Steel Materials

The yield strength, ultimate tensile strength, and elastic modulus of Q345 were 361.3 MPa, 479.6 MPa, and 200.3 GPa, respectively. The yield strength, ultimate tensile strength, and elastic modulus of Q420 were 449.6 MPa, 600.2 MPa, and 201.5 GPa, respectively. HRB400 was used for the reinforcing bars, and the yield strength, ultimate tensile strength, and elastic modulus of HRB400 were 439.3 MPa, 577.1 MPa, and 203.7 GPa, respectively.

**Figure 11:**
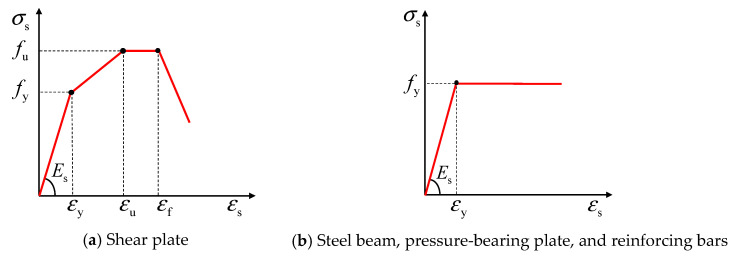
Stress–strain relationship of steel.

Figure 11 presents the stress–strain relationship for steel. As shown in Figure 11a, the descending branch of the stress–strain curve of the shear plate was used to simulate shear-plate failure [27,30]. The ultimate strain *ε*_u_ and fracture strain *ε*_f_ of the shear plate used in the FE model were 0.13 and 0.135, respectively. As shown in Figure 11b, the ideal elastoplastic bilinear model was adopted to model other steel components except the shear plate [30,31].

## 4. Verification of the Numerical Model

### 4.1. Comparison of Shear Resistance and Slip Modulus

The results of push-out tests were used to validate the effectiveness of the numerical model. A comparison between the ultimate shear resistance and slip modulus of the B-SCs in tests and numerical analysis, listed in Table 1, shows a high agreement for all push-out specimens, with a maximum deviation of 6% found for B-SC-r20-h160-3. The mean value of $P_{u,\;\mathrm{test}}/P_{u,\;\mathrm{FE}}$ was 0.99 with a standard deviation of 0.03 and the mean value $K_{0.2,\;\mathrm{test}}/K_{0.2,\;\mathrm{FE}}$ was 1.00 with a standard deviation of 0.03. This demonstrates that the established numerical model can effectively perform a parametric study on the shear resistance and slip modulus of the B-SCs.

### 4.2. Comparison of Load-Slip Curves

**Figure 12:**
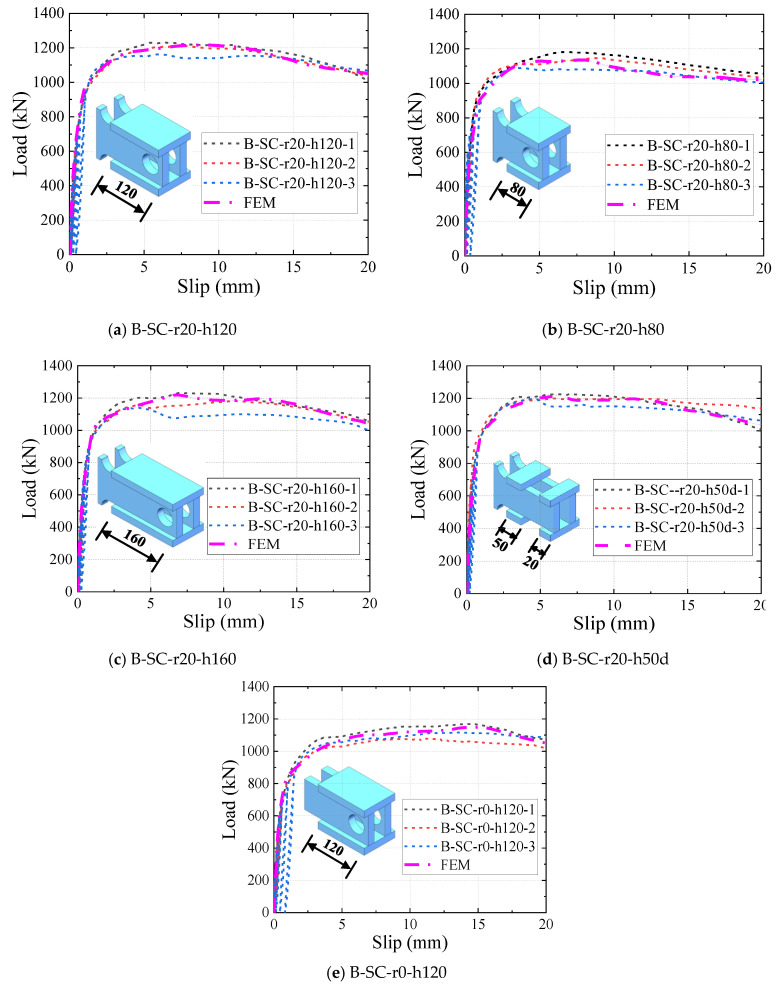
Comparison of load-slip curves.

As shown in Figure 12, the load-slip curves derived from the FE model were observed to be in close agreement with the push-out tests. Figure 12 also reveals that the load-slip curves of all push-out specimens followed a similar trend, and they can be divided into three distinct stages. At the initial elastic stage, the shear load increased rapidly with little slip, indicating that the B-SCs had a high slip modulus at the initial stage. Subsequently, the slip increased rapidly, while the shear load slowly increased to the peak load. Finally, the load gradually decreased as the slip continued to increase. Therefore, the FE model can effectively evaluate the overall trend of the load-slip curves of the B-SCs.

### 4.3. Comparison of Failure Modes

**Figure 13:**
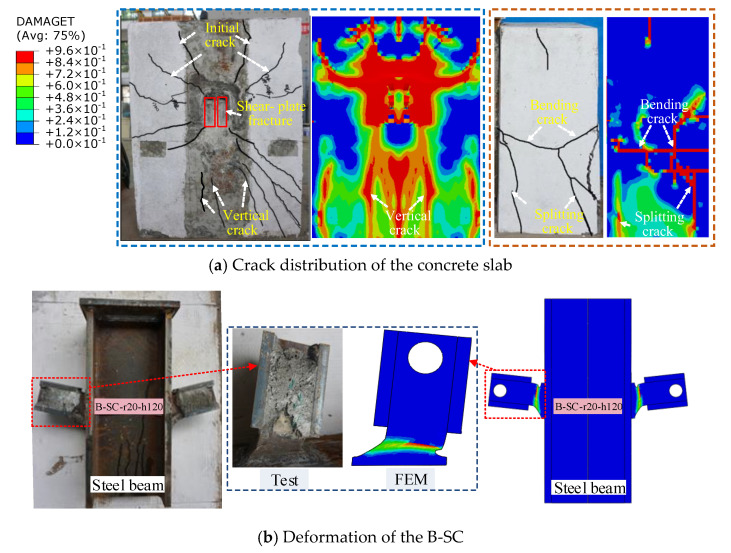
Failure modes of the specimen B-SC-r20-h120.

As shown in Figure 13, the failure modes derived from the FE model closely matched those observed in push-out tests. The distribution and development law of the concrete-slab cracks in the FE model matched well with the experimental response. As illustrated in Figure 13b, the shear plate exhibited significant shear deformation along the loading direction in both the experiments and FE modelling, whereas the pressure-bearing plate exhibited no evident deformation. According to the studies reported above, the FE model can accurately predict the shear behaviour of the B-SCs.

### 4.4. Failure Process of B-SCs

In Section 4.3, the failure modes of the push-out specimens were primarily proved as splitting and shear failures of the concrete slab and shear plate, respectively. In addition, push-out tests have demonstrated that shear deformation of the shear plate provides the majority of the shear resistance of the B-SCs [25]. Therefore, an analysis of the failure mechanisms of concrete slabs and shear plates is necessary.

**Figure 14:**
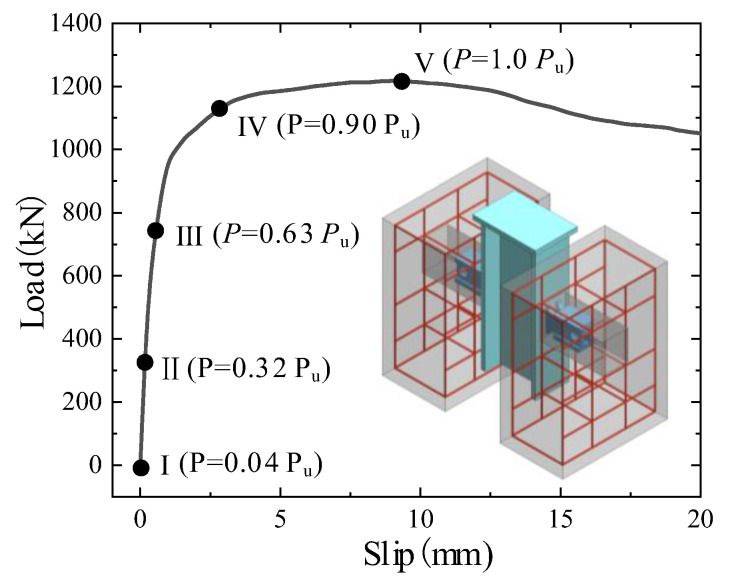
Load-slip curve of the specimen B-SC-r20-h120 (FE model).

**Figure 15:**
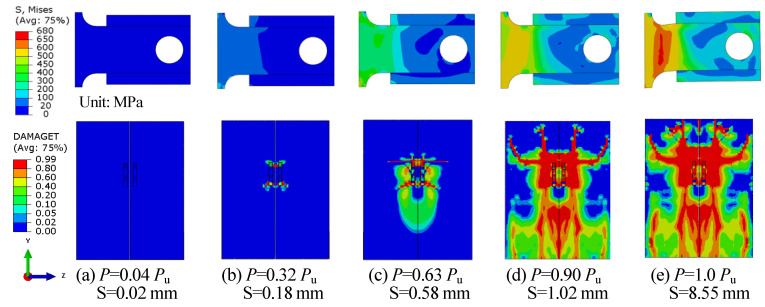
Deformation and stress distribution of concrete slab and shear plate.

According to the B-SC-r20-h120 FE model, the complete failure process of the B-SCs was analysed as follows. The load-slip curve for specimen B-SC-r20-h120 is shown in Figure 14, on which five typical points are marked, with points I and II representing the elastic stage and points III and IV representing the elastic–plastic stage, and point V representing the ultimate state [42]. Figure 15 presents the deformation of the concrete slab and the stress of the shear plate at these five key points, which illustrates the complete failure process of the push-out specimens in detail.

When *P* = 0.04 *P*_u_ and slip = 0.02 mm, the specimen exhibited the elastic stage. As shown in Figure 15a, the shear-plate stress did not exceed 20 MPa, and no cracks appeared in the concrete slab.

When *P* = 0.32 *P*_u_ and slip = 0.18 mm, the specimen still exhibited an elastic response. The stress in the anchorage and shear zones of the B-SC was significantly greater than that in other areas, as illustrated in Figure 15b. In addition, the concrete slab at the root of the B-SC exhibited small cracks owing to the extrusion of the shear plate, which is consistent with the phenomenon of splitting cracks in the concrete slab owing to the extrusion of the stud connector [11].

When *P* = 0.63 *P*_u_ and slip = 0.58 mm, plastic deformation occurred locally in the push-out specimen. As shown in Figure 15c, the local stress in the anchorage and shear zones of the B-SC was larger than the yield strength owing to the combined action of bending and shear. The existing cracks in the concrete slab near the B-SC continued to extend under the action of the load and the cracked area extended from the post-poured high-strength mortar to the precast concrete slab.

When *P* = 0.90 *P*_u_ and slip = 2.27 mm, as shown in Figure 15d, the stress in the shear-plate zone was greater than the yield strength. Cracks in the concrete slab developed significantly and cracks near the B-SC extended to the bottom and top of the concrete slab.

When *P* = 1.0 and slip = 8.55 mm, the specimen reached its ultimate state. The local stress in the shear zone of the B-SC reached the ultimate tensile strength of steel, as shown in Figure 15e. The stress in the anchorage zone was less than that in the shear zone, which ensured that the failure of the shear zone of the B-SC preceded that of the anchorage zone. At this time, the cracks spread throughout the precast concrete slab, indicating that the B-SC reached the ultimate state, mainly because the cracked concrete slab was insufficient to support the increase in load.

## 5. Parametric Study

**Figure 16:**
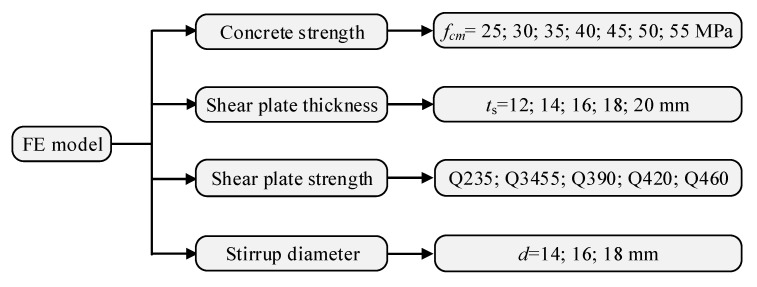
FE models evaluated in parametric study.

The effect of the following factors on the shear behaviour of the B-SCs were investigated in the parametric study: concrete strength, the thickness and the tensile strength of the shear plate, and stirrup diameter, as shown in Figure 16.

### 5.1. Effect of Concrete Strength

**Figure 17:**
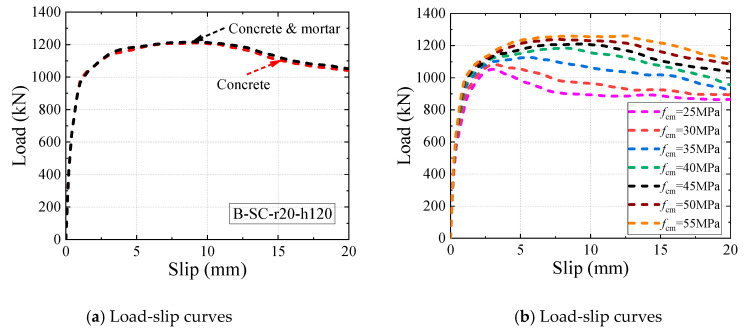

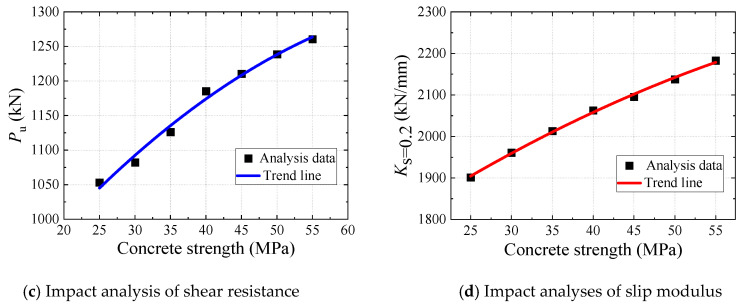
Effect of concrete strength.

As shown in Figure 17a, when the numerical model included and excluded the postpoured high-strength mortar, the two load-slip curves almost coincided, which was consistent with Yu’s conclusion [43]. For conservatism and simplicity, high-strength mortar was not included in the subsequent parametric analysis. A comparison of the load-slip curves for different concrete strengths (25 MPa–55 MPa) [36] is illustrated in Figure 17b. As shown in Figure 17b,c, the ultimate shear resistance and slip modulus of the B-SCs increased as the concrete strength increased. According to Figure 17b, the ductility of the B-SCs also increased as the concrete strength increased, which confirmed the conclusion of Oehlers [44].

**Figure 18:**
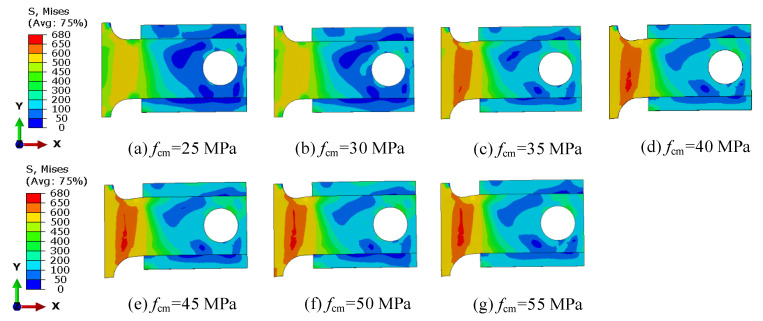
Stress-cloud diagram of B-SCs on the ultimate state (MPa).

Figure 18 presents the stress distribution of the B-SCs for different concrete strengths in the ultimate state. When the concrete strength increased, the shear deformation and high-stress area of the shear plate in the ultimate state also increased. This can be attributed to the increase in the cracking resistance of a precast concrete slab with the increase in concrete strength. Higher-strength concrete enables concrete to support greater shear deformation of the shear plate, which increases the contribution of the shear plate to the shear resistance of B-SCs, thereby improving the ultimate shear resistance of B-SCs.

### 5.2. Effect of Shear-Plate Thickness

**Figure 19:**
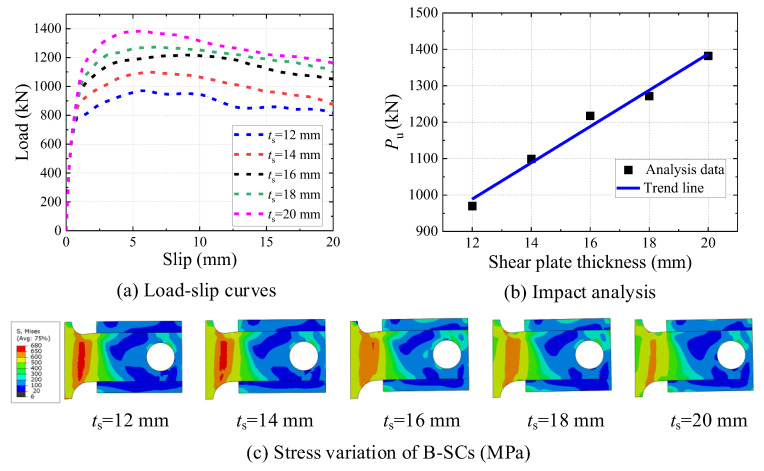
Effect of shear-plate thickness.

Previous experimental studies have demonstrated a significant impact of the diameter of studs and bolts on their ultimate shear resistance [45,46]. Similarly, Table 2 demonstrates the significant impact of shear-plate thickness on the ultimate shear resistance and but a negligible impact on the slip modulus of the B-SCs. As summarized in Table 2, the ultimate shear resistance and slip modulus increased by 42.5% and 2.2%, respectively, when the shear-plate thickness increased from 12 to 20 mm. As illustrated in Figure 19b, the ultimate shear resistance increased approximately linearly with the shear plate thickness. Figure 19c presents the stress-cloud diagram of the shear plates for different thicknesses in the ultimate state. The shear deformation and high-stress area of the shear plate can be observed to have decreased as the shear-plate thickness increased. This may be attributable to the fact that increasing the shear-plate thickness can increase the effective shear-cross area of the shear plate, thereby lowering its shear deformation, increasing the bearing area of concrete, and improving the ultimate shear resistance of B-SCs.

### 5.3. Effect of Shear-Plate Tensile Strength

According to GB 50017-2017 [32], five types of structural steel were selected for the parametric analysis, as listed in Table 2.

**Figure 20:**
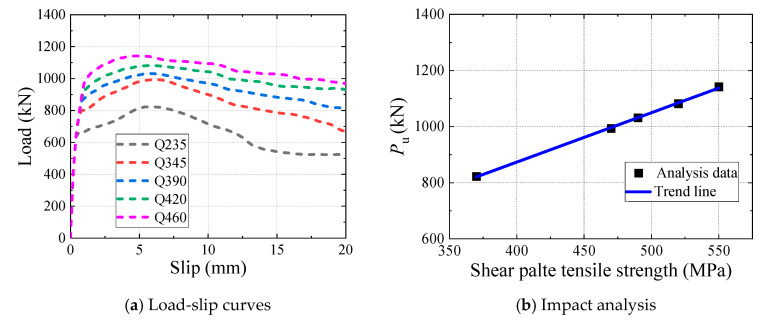
Effect of shear-plate tensile strength.

Figure 20 presents the effect of the shear-plate tensile strength on the shear behaviour of the B-SCs, and a comparison between the ultimate shear resistance and slip modulus of the specimens is summarised in Table 2. As depicted in Figure 20b, the ultimate shear resistance increased approximately linearly with the shear-plate tensile strength, whereas the slip modulus remained constant because the elastic modulus of steels with varying tensile strengths were nearly identical. The ultimate shear resistance increased by 38.8% when the steel type of the shear plate was changed from Q235 to Q460, indicating the significant impact of the shear-plate tensile strength on the ultimate shear resistance of the B-SCs.

### 5.4. Effect of Stirrup Diameter

**Figure 21:**
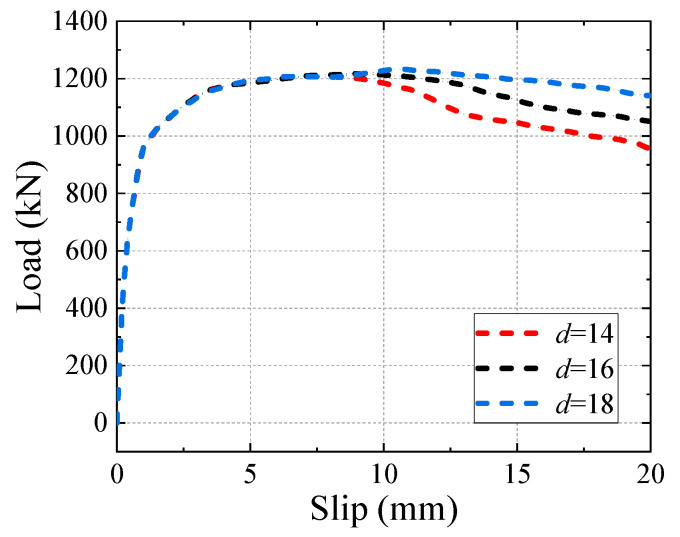
Effect of stirrup diameter.

The confinement of stirrup to concrete slabs has been demonstrated to significantly affect the behaviour of shear connectors [42,47]. Figure 21 presents the effect of the stirrup diameter on the shear behaviour of the B-SCs and a comparison between the ultimate shear resistance and slip modulus is summarised in Table 3. The ultimate shear resistance improved by 1.7% when the stirrup diameter *d* increased from 14 to 18 mm, but the slip modulus remained constant because the stirrup had not imposed its confinement action on concrete when the relative slip was 0.2 mm.

## 6. Proposed Shear-Calculation Formulae and Validation

### 6.1. Proposed Formula for Predicating Ultimate Shear Resistance

**Figure 22:**
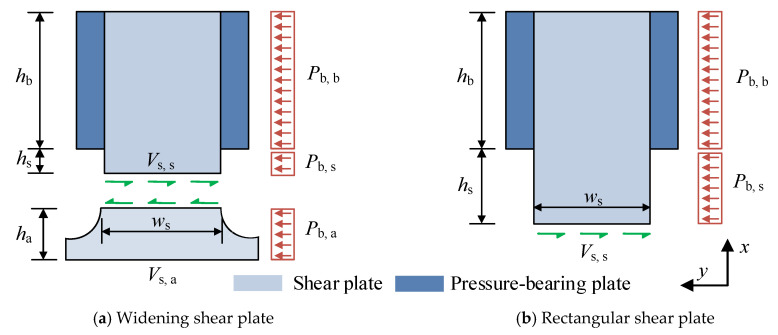
Shear mechanism of B-SCs.

According to Section 4.3 and Section 4.4, the failure modes of the push-out specimens with B-SCs were primarily the splitting and shear failures of the concrete slab and shear plate, respectively, indicating the primarily affected shear resistance of the B-SCs by the properties of the concrete slab and shear plate. Figure 22 presents the shear mechanism of the B-SCs. A parametric study showed that the concrete strength (*f*_cm_), the thickness (*t*_s_), and the tensile strength (*f*_u_) of the shear plate had a significant impact on the ultimate shear resistance. Especially, the concrete strength directly affects the shear deformation of the shear plate. Therefore, introducing a coefficient $\lambda_{s}\left(=\alpha \frac{E_{\mathrm{cm}}}{f_{\mathrm{cm}}}+\beta \right)$ was reasonable, which is related to the concrete-strength grade, to quantify the contribution of the shear plate to the ultimate shear resistance; *α* and *β* are the coefficients. Therefore, the new design formula for the ultimate shear resistance of B-SCs was suggested as follows [25]:

(12) $$P_{u}=V_{s,s}+P_{b,a}$$

(13) $$V_{s,s}=\lambda_{s}A_{s,s}f_{u}$$

(14) $$P_{b,a}=\lambda_{b}A_{b,a}\sqrt{E_{\mathrm{cm}}f_{\mathrm{cm}}}$$

Based on the results of tests and FE parametric study, the values of *α* and *β* are 0.0003 and 0.82, respectively, when the least-squares method is used. In Equation (12), *V*_s,s_ is the shear capacity of the shear zone and *P*_b,a_ is the compressive capacity of concrete in the anchorage zone. In Equation (13), *A*_s,s_ (*t*_s_*w*_s_) is the cross-sectional area of the shear zone, and *t*_s_, *w*_s_, and *f*_u_ are the thickness, width, and tensile strength of the shear plate, respectively. *λ*_b_ (=0.052) is a constant coefficient [25], and *A*_b,a_ is the local bearing area of the anchoring zone. *f*_cm_ and *E*_cm_ are the compressive cylinder strength and elastic modulus of concrete, respectively.

**Figure 23:**
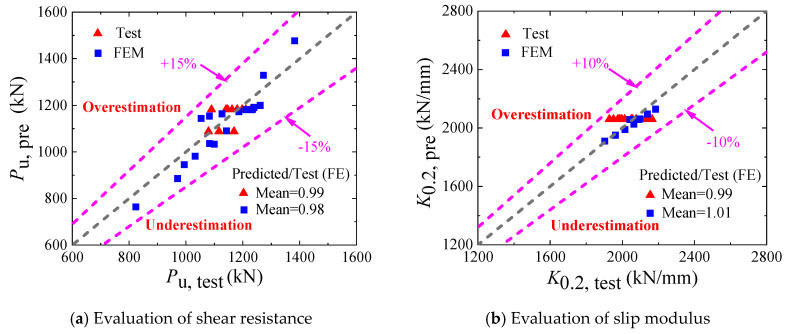
Validation of the proposed design formulae.

The proposed ultimate shear-resistance formula for B-SCs was validated by comparing it with the results of experiments and FE parametric analysis in Table 4 and Table 5, and Figure 23a. The mean value of $P_{u,\;\mathrm{pre}}/P_{u,\;\mathrm{test}}$ was 0.99 with a standard deviation of 0.037; the mean value of $P_{u,\;\mathrm{pre}}/P_{u,\;\mathrm{FEM}}$ was 0.98 with a standard deviation of 0.048, indicating that the proposed design formula can accurately predict the ultimate shear resistance of B-SCs.

### 6.2. Proposed Formula for Predicating Slip Modulus

The slip modulus of B-SCs is determined by the secant slope of the load-slip curves at a slip of 0.2 mm [48,49,50]. The advantage of this method is the fixed-slip value, and the calculation error caused by the relative dispersion of the test values of the ultimate shear resistance can be avoided. Shim [9] proposed a Formula (15) to predict the slip modulus of large-diameter studs. The formula considers the effect of concrete strength on slip modulus.

(15) $$k_{s}=\frac{P_{\max}}{d(0.16-0.0017f_{c})}$$

In JTG/T D64-01-2015 [51], the slip modulus of studs was expressed as Equation (16), which accounts for the contributions of the concrete elastic modulus, concrete strength, and stud diameter to the slip modulus. Hu [50] proposed a slip modulus design formula for large-diameter stud connectors, as expressed by Equation (17).

(16) $$k_{s}=13.0d\sqrt{E_{c}f_{c}}$$

(17) $$k_{s}=0.62d^{2}\sqrt{E_{c}f_{c}}$$

According to the parametric analysis, concrete strength directly affected the failure modes of the push-out specimens. Figure 17d shows a significant effect of concrete strength on slip modulus, while the shear-plate thickness had a minor effect on slip modulus. Therefore, the influence of the concrete strength on the slip modulus was quantified by introducing a parameter *λ*_k_ related to the concrete strength. A new formula for the slip modulus of B-SCs was proposed by regression analysis as follows:

(18) $$K_{0.2}=(0.0016\frac{E_{\mathrm{cm}}}{f_{\mathrm{cm}}}+0.37)\sqrt{E_{\mathrm{cm}}f_{\mathrm{cm}}}$$

In Equation (18), $\lambda_{k}=0.0016E_{\mathrm{cm}}/f_{\mathrm{cm}}$. The proposed slip-modulus formula was validated by comparing the tests and FE analysis results in Table 4 and Table 5, and Figure 23b. The mean value of $K_{0.2,\;\mathrm{pre}}/P_{0.2,\;\mathrm{test}}$ was 0.99 with a standard deviation of 0.035, and the mean value of $K_{0.2,\;\mathrm{pre}}/P_{0.2,\;\mathrm{FE}}$ was 1.01 with a standard deviation of 0.008, indicating that the proposed design formula can accurately evaluate the slip modulus of B-SCs.

## 7. Conclusions

In this study, a three-dimensional refined nonlinear numerical model was established to investigate the shear behaviour of B-SCs. Then, using the verified numerical model, the effects of the concrete strength and the thickness and the tensile strength of the shear plate on the shear behaviour of B-SCs were investigated. The following conclusions can be drawn:

1. The numerical model matched well with the push-out tests in terms of the ultimate shear resistance, slip modulus, load-slip curves, and failure modes, indicating the accurate evaluation of the shear behaviour of B-SCs in prefabricated composite structures using the numerical model;
2. All push-out specimens with B-SCs exhibited mixed failure modes composed of splitting and shear failures of the concrete slab and shear plate, respectively. The higher strength concrete enables concrete to support greater shear deformation of the shear plate, which increases the contribution of the shear plate to the shear resistance of B-SCs, thus improving the ultimate shear resistance of B-SCs;
3. The ultimate shear resistance of B-SCs increased approximately linearly with the increase in the thickness and the tensile strength of the shear plate because the shear resistance of B-SCs was mainly determined by the shear resistance of the shear plate. However, these two parameters had a minor influence on the slip modulus of B-SCs;
4. New calculation formulae for the ultimate shear resistance and slip modulus of the B-SCs were proposed. Both formulae, which accounted for the effect of concrete strength on the shear deformation of the shear plate, agreed well with the results of the push-out tests and numerical analysis.

## Figures and Tables

**Table 1:** Comparison between experimental results and FE results.

Specimens	*P*_u,test_	*P*_u,FE_	*K*_0.2,test_	*K*_0.2,FE_	$\frac{P_{u,\;\mathrm{test}}}{P_{u,\;\mathrm{FE}}}$	$\frac{K_{0.2,\;\mathrm{test}}}{K_{0.2,\;\mathrm{FE}}}$
	(kN)	(kN)	(kN/mm)	(kN/mm)		
B-SC-r20-h120-1	1230.0	1217.4	2076.2	2073.7	1.01	1.00
B-SC-r20-h120-2	1210.5		2013.7		0.99	0.97
B-SC-r20-h120-3	1162.2		1997.6		0.95	0.96
B-SC-r20-h80-1	1180.9	1135.8	1987.3	2008.2	1.04	0.99
B-SC-r20-h80-2	1146.2		2056.6		1.01	1.02
B-SC-r20-h80-3	1089.6		2051		0.96	1.02
B-SC-r20-h160-1	1231.5	1219.0	2051.2	2060.7	1.01	1.00
B-SC-r20-h160-2	1180.1		1984.7		0.97	0.96
B-SC-r20-h160-3	1140.9		1927.9		0.94	0.94
B-SC-r20-h50d-1	1228.1	1216.7	2142.3	2075.7	1.02	1.03
B-SC-r20-h50d-2	1198		2165.0		0.94	1.04
B-SC-r20-h50d-3	1197.6		2125.1		0.97	1.02
B-SC-r0-h120-1	1168.8	1149.2	1975.4	1940.0	1.01	1.02
B-SC-r0-h120-2	1079.2		1928.7		0.98	0.99
B-SC-r0-h120-3	1116.1		1951.6		0.98	1.01
Mean					0.99	1.00
Standard deviation					0.03	0.03

where *P*_u_ is the ultimate shear resistance and *K*_0.2_ is the secant slope of the load-slip curves at a slip of 0.2 mm.

**Table 2:** Results of FE parametric study.

Specimens	*E*_s_ (GPa)	*f*_y_ (MPa)	*f*_u_ (MPa)	Shear-Plate Thickness (mm)	*P*_u,FEM_ (kN)	*K*_0.2,FEM_ (kN/mm)
B-SC-shear-12	201.5	449.6	600.2	12	969.9	2041.2
B-SC-shear-14				14	1099.5	2058.6
B-SC-shear-16				16	1217.4	2073.4
B-SC-shear-18				18	1271.7	2077.6
B-SC-shear-20				20	1381.9	2086.7
B-SC-Q235	210	235	370	16	822.9	2073.4
B-SC-Q345	210	345	470		993.5	2073.4
B-SC-Q390	210	390	490		1030.6	2073.4
B-SC-Q420	210	420	520		1081.8	2073.4
B-SC-Q460	210	460	550		1142.2	2073.4

**Table 3:** Results of FE parametric analysis.

Specimen	*d*	*P*_u_	*K*_0.2_
	mm	(kN)	(kN/mm)
B-SC-stirrup-14	14	1212.3	2070.3
B-SC-stirrup-16	16	1217.4	2070.3
B-SC-stirrup-18	18	1232.9	2070.3

**Table 4:** Comparison between predicted values and tests.

Tests Specimens	*P*_u,test_	*P*_u,pre_	*K*_0.2,test_	*K*_0.2,pre_	$\frac{P_{u,\;\mathrm{pre}}}{P_{u,\;\mathrm{test}}}$	$\frac{K_{0.2,\;\mathrm{pre}}}{K_{0.2,\;\mathrm{test}}}$
	(kN)	(kN)	(kN/mm)	(kN/mm)		
B-SC-r20-h120-1	1230.0	1182.0	2076.2	2059.2	0.96	1.01
B-SC-r20-h120-2	1210.5	1182.0	2013.7	2059.2	0.98	0.98
B-SC-r20-h120-3	1162.2	1182.0	1997.6	2059.2	1.02	0.97
B-SC-r20-h80-1	1180.9	1182.0	1987.3	2059.2	1.00	0.97
B-SC-r20-h80-2	1146.2	1182.0	2056.6	2059.2	1.03	1.00
B-SC-r20-h80-3	1089.6	1182.0	2051	2059.2	1.08	1.00
B-SC-r20-h160-1	1231.5	1182.0	2051.2	2059.2	0.96	1.00
B-SC-r20-h160-2	1180.1	1182.0	1984.7	2059.2	1.00	0.96
B-SC-r20-h160-3	1140.9	1182.0	1927.9	2059.2	1.04	0.94
B-SC-r20-h50d-1	1228.1	1182.0	2142.3	2059.2	0.96	1.04
B-SC-r20-h50d-2	1198	1182.0	2165.0	2059.2	0.99	1.05
B-SC-r20-h50d-3	1197.6	1182.0	2125.1	2059.2	0.99	1.03
B-SC-r0-h120-1	1168.8	1088.0	1975.4	2059.2	0.93	0.96
B-SC-r0-h120-2	1079.2	1088.0	1928.7	2059.2	1.01	0.94
B-SC-r0-h120-3	1116.1	1088.0	1951.6	2059.2	0.97	0.95
Mean					0.99	0.99
Standard deviation					0.037	0.035

**Table 5:** Comparison between predicted values and FE analysis.

Parametric Study	Specimens	*P*_u,FE_	*P*_u,pre_	*K*_0.2,test_	*K*_0.2,pre_	$\frac{P_{u,\;\mathrm{pre}}}{P_{u,\;\mathrm{FE}}}$	$\frac{K_{0.2,\;\mathrm{pre}}}{K_{0.2,\;\mathrm{FE}}}$
		(kN)	(kN)	(kN/mm)	(kN/mm)		
Concrete strength	B-SC-*f*_cm_-25	1053.0	1144.3	1901.7	1911.1	1.09	1.00
	B-SC-*f*_cm_-30	1082.2	1154.6	1961.2	1951.9	1.07	1.00
	B-SC-*f*_cm_-35	1126.3	1164.3	2013.4	1990.5	1.03	1.01
	B-SC-*f*_cm_-40	1185.3	1173.7	2062.8	2027.4	0.99	1.02
	B-SC-*f*_cm_-45	1210.6	1182.9	2095.3	2062.7	0.98	1.02
	B-SC-*f*_cm_-50	1238.8	1191.7	2137.9	2096.7	0.96	1.02
	B-SC-*f*_cm_-55	1260.7	1200.3	2183.0	2129.6	0.95	1.03
Shear-plate thickness	B-SC-shear-12	969.9	886.5	2041.2	2059.2	0.91	0.99
	B-SC-shear-14	1099.5	1034.2	2058.6	2059.2	0.94	1.00
	B-SC-shear-16	1217.4	1182.0	2073.4	2059.2	0.97	1.01
	B-SC-shear-18	1271.8	1329.7	2077.6	2059.2	1.05	1.01
	B-SC-shear-20	1382.0	1477.4	2086.7	2059.2	1.07	1.01
Shear-plate tensile strength	B-SC-Q235	823.0	764.9	2070.3	2059.2	0.93	1.01
	B-SC-Q345	993.5	946.2	2070.3	2059.2	0.95	1.01
	B-SC-Q390	1031.6	982.5	2070.3	2059.2	0.95	1.01
	B-SC-Q420	1081.8	1036.9	2070.3	2059.2	0.96	1.01
	B-SC-Q460	1142.2	1091.3	2070.3	2059.2	0.96	1.01
Stirrup diameter	B-SC-stirrup-14	1212.3	1182.0	2070.3	2059.2	0.97	1.01
	B-SC-stirrup-16	1217.4	1182.0	2070.3	2059.2	0.97	1.01
	B-SC-stirrup-18	1232.9	1182.0	2070.3	2059.2	0.96	1.01
Mean						0.98	1.01
Standard deviation						0.048	0.008

## Data Availability

The data presented in this study are available on request from the corresponding author.

## Abbreviations

$\delta_{n}^{f}$	Maximum separation of cohesive-contact property
$\varepsilon_{c}^{\mathrm{ch}}$	Crushing strain of concrete
$\varepsilon_{t}^{\mathrm{ck}}$	Tensile cracking strain of concrete
*A*_b,a_	Local bearing area of the anchorage zone
*A*_s,s_	Cross-sectional area of the shear zone
*b*	$\varepsilon_{c}^{\mathrm{pl}}/\varepsilon_{c}^{\mathrm{in}}$ ratio in Equation (6)
*c*_1_ and *c*_2_	Coefficients in Equation (7)
*d*	Stirrup diameter
*d*_c_	Concrete compression-damage variable
*d*_s_	Stud diameter in Equation (16)
*d*_t_	Concrete tensile-damage variable
*E*_0_	Undamaged elastic modulus of concrete
*E*_cm_	Elastic modulus of concrete
*E*_s_	Elastic modulus of steel
*f*_c_	Concrete strength in Equations (16)–(18)
*f*_cm_	Cylinder compressive strength of concrete
*f*_tm_	Tensile strength of concrete
*f*_u_	Ultimate tensile strength of steel
*f*_y_	Yield strength of steel
*G*_ch_	Crushing energy per unit area of concrete
*G*_cm_	Shear modulus of concrete
*G*_f_	Crushing energy per unit area of concrete
*h*_b_/*h*_s_/*h*_a_	Height of the pressure-bearing zone/shear zone/anchorage zone
*K*	Second stress invariant ratio
*K*_0.2_	Slip modulus (secant slope of the load-slip curves at a slip of 0.2 mm.)
*K*_0.2,FE_	Slip modulus obtained from finite element
*K*_0.2,pre_	Predicted slip modulus
*K*_0.2,test_	Slip modulus obtained from test
*K*_nn_, *K*_ss_, *K*_tt_	Elastic stiffness of cohesive contact property
*k_s_*	Slip modulus of stud shear connector in Equation (16)
*l*_ck_	Characteristic element length
Nomenclature	
*P*	Shear load
*P*_b,a_	Compressive capacity of concrete in the anchorage zone
*P*_b,b_	Compressive capacity of concrete in the pressure-bearing zone
*P*_b,s_	Compressive capacity of concrete in the shear zone
*P*_max_	Ultimate shear resistance of stud shear connector in Equation (16)
*P*_u_	Ultimate shear resistance
*P*_u,FE_	Ultimate shear resistance obtained from finite element
*P*_u,pre_	Predicted ultimate shear resistance
*P*_u,test_	Ultimate shear resistance obtained from test
*S*	Relative ship
*t*_n_, *t*_s_, *t*_t_	Tractions of the cohesive contact property
*t*_s_, *w*_s_	Thickness and width of the shear plate in the shear zone
*V*_s,a_	Shear capacity of the anchorage zone
*V*_ss_	Shear capacity of the shear zone
*w*	Cracking width of concrete
*w*_c_	Cracking width corresponding to the zero tensile stress
*α*_c_/*α*_t_/*b*_c_/*b*_t_	Dimensionless coefficients in Equations (8)–(11)
*α*_E_	Constant factor about concrete aggregates
*ε*	Flow potential eccentricity
*ε*_c_	Compressive strain of concrete
*ε*_tm_	Tensile peak strain of concrete
*λ*_b_	Constant coefficient in Equation (14)
*λ*_s_, *α* and *β*	Constant coefficient in Equation (13)
*μ*	Viscosity parameter
*σ*_bo_/*σ*_co_	Ratio of biaxial to uniaxial compressive strength
*σ*_c_	Compressive stress of concrete
*σ*_t_	Tensile stress of concrete
*ψ*	Dilatancy angle
